# 722 Collagen-elastin matrix for defect coverage in exposed bradytrophic tissue after severe burn and degloving injuries

**DOI:** 10.1093/jbcr/irac012.276

**Published:** 2022-03-23

**Authors:** Markus Oehlbauer

**Affiliations:** BG Trauma Center Murnau, Murnau, Bayern

## Abstract

**Introduction:**

Despite successful defect coverage by means of complex skin or muscle flaps, particularly large and deep problematic wounds with exposed bradytrophic tissues after soft tissue loss are very susceptible to surgical revision.

A dermal matrix, consisting of native collagen (collagen type I, III and V) supplemented by an elastin hydolyzate was first used for the treatment of burns, predominantly those that were full-thickness. Subsequently, its use was extended to defect coverage especially after soft tissue loss caused by degloving injuries.

**Methods:**

52 patients with exposed bradytrophic tissues caused by severe burn or degloving injuries were treated the same way.

In all patients operative debridement showed soft tissue loss with free bone or free periostal structures, exposed tendons or joint capsules.

In all patients after accurate wound bed preparation defect coverage was performed with collagen-elastin matrix and unmeshed split skin grafts in combination with negative pressure wound therapy for fixation of dermal matrix and split skin grafts.

**Results:**

Two-years follow up of these collagen-elastin matrix procedures in defect coverage showed an excellent functional outcome:

Up until now, no areas with unstable scars have occurred, no surgical scar revisions were required. The patients were still able to wear normal footwear, clinical gait analysis showed perfect functional outcome.

**Conclusions:**

The application of collagen-elastin matrix in patients with exposed bradytrophic tissues after severe burn or degloving injury treated so far represents an excellent reconstruction method, from initial coverage to scar development.

